# Supplementary material for: Investigating the influence of physiologically relevant hydrostatic pressure on CHO cell batch culture
Source: Sci Rep. 2021 Jan 8;11:162. doi: 10.1038/s41598-020-80576-8 (PMC7794228; doi:10.1038/s41598-020-80576-8)
Supplement: Supplementary file 1 — Supplementary Information 1. [file 41598_2020_80576_MOESM1_ESM.zip › S1/IgG/Feb14 0 30 60 90/90mmHg Feb10.pdf]

Data File C:\Users\P...mStation\1\Data\TK_CHO_IgG 2019-02-14 14-17-55\018-10-ML 90 Feb10.D
Sample Name: ML 90 Feb10

===

Acq. Operator : SYSTEM Seq. Line : 18
Sample Operator : SYSTEM
Acq. Instrument : HPLC-0039 Location : 10
Injection Date : 2/14/2019 3:32:15 PM Inj : 1
Inj Volume : 100.000 µl
Method : C:\Users\Public\Documents\ChemStation\1\Data\TK_CHO_IgG 2019-02-14 14-17-55 \CHO_IgG_LC.M (Sequence Method)
Last changed : 2/8/2019 3:17:23 PM by SYSTEM

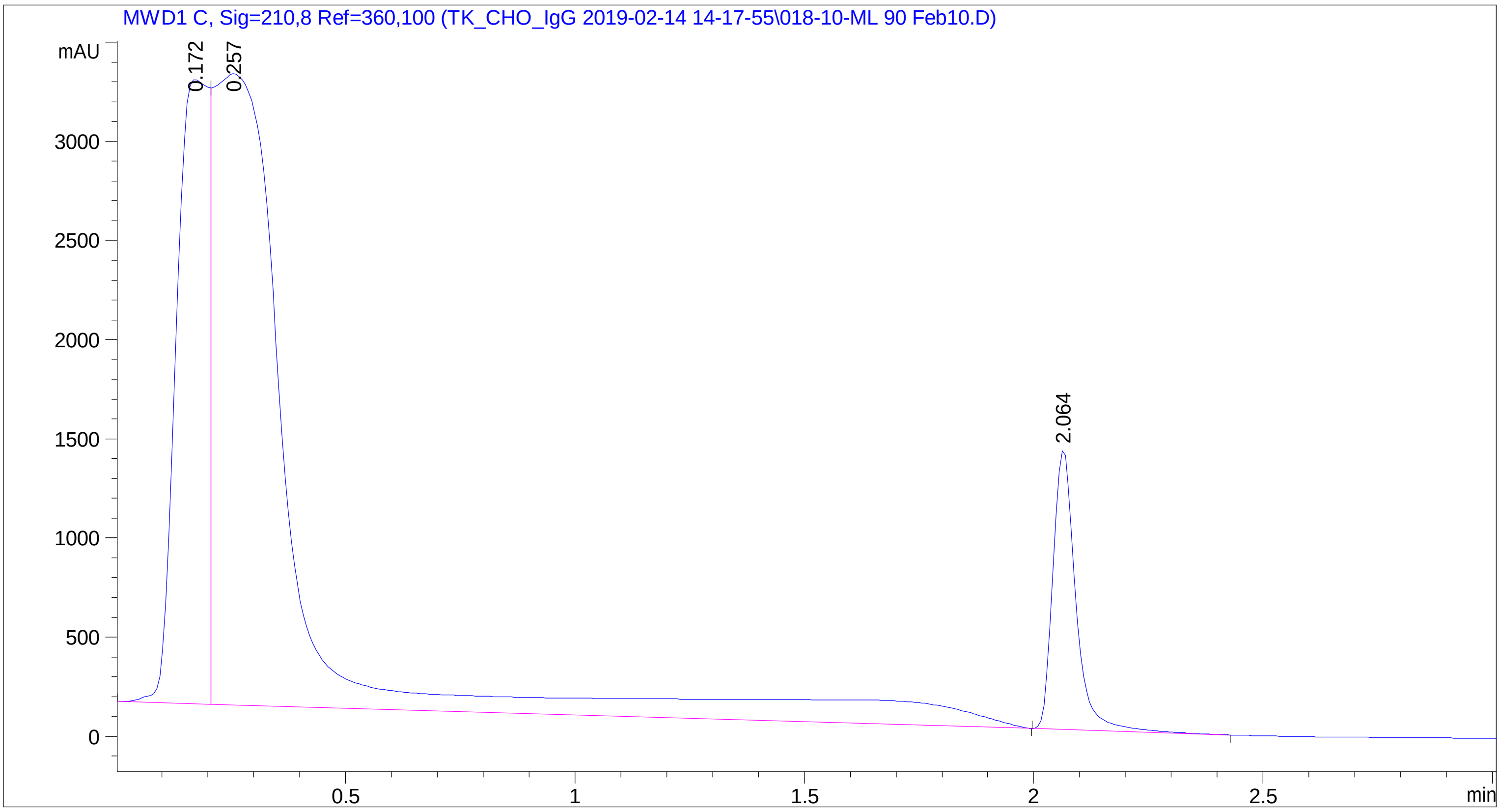

===

Area Percent Report

===

Sorted By : Signal
Multiplier : 1.0000
Dilution : 1.0000
Use Multiplier & Dilution Factor with ISTDs

Signal 1: MWD1 C, Sig=210,8 Ref=360,100

Peak #	RetTime [min]	Type	Width [min]	Area [mAU*s]	Height [mAU]	Area %
1	0.172	BV	0.0744	1.52111e4	3146.79565	26.0352
2	0.257	VB	0.1724	3.83091e4	3182.72656	65.5697
3	2.064	BBA	0.0545	4904.79541	1413.79797	8.3950

Totals : 5.84250e4 7743.32019

===

*** End of Report ***
